# Improving the Carotenoid Content in Maize by Using Isonuclear Lines

**DOI:** 10.3390/plants11131632

**Published:** 2022-06-21

**Authors:** Roxana Elena Calugar, Edward Muntean, Andrei Varga, Carmen Daniela Vana, Voichita Virginia Has, Nicolae Tritean, Loredana Anca Ceclan

**Affiliations:** 1 Agricultural Research and Development Station Turda, Agriculturii 27, 401100 Turda, Romania; roxana.calugar@scdaturda.ro (R.E.C.); andrei_varga06@yahoo.com (A.V.); carmend.vana@yahoo.com (C.D.V.); hasvoichita@yahoo.com (V.V.H.); ancaceclan@gmail.com (L.A.C.); 2 Department of Food Science, Faculty of Food Science and Technology, University of Agricultural Sciences and Veterinary Medicine Cluj-Napoca, 3-5 Mănăştur St., 400372 Cluj-Napoca, Romania

**Keywords:** carotenoids, maize, isonuclear lines, hybrids

## Abstract

Carotenoids are important biologically active compounds in the human diet due to their role in maintaining a proper health status. Maize (*Zea mays* L.) is one of the main crops worldwide, in terms of production quantity, yield and harvested area, as it is also an important source of carotenoids in human nutrition worldwide. Increasing the carotenoid content of maize grains is one of the major targets of the research into maize breeding; in this context, the aim of this study was to establish the influence of some fertile cytoplasm on the carotenoid content in inbred lines and hybrids. Twenty-five isonuclear lines and 100 hybrids were studied for the genetic determinism involved in the transmission of four target carotenoids: lutein, zeaxanthin, β-cryptoxanthin and β-carotene. The analysis of carotenoids was carried out using high performance liquid chromatography using a Flexar system with UV-VIS detection. The obtained data revealed that the cytoplasms did not have a significant influence on the carotenoid content of the inbred lines; larger differences were attributed to the cytoplasm × nucleus interaction. For hybrids, the cytoplasmic nuclear interactions have a significant influence on the content of lutein, zeaxanthin and β-cryptoxanthin. For the cytoplasm × nucleus × tester interactions, significant differences were identified for all traits.

## 1. Introduction

Maize (*Zea mays*) is one of the main crops worldwide in terms of production quantity, yield and harvested area; between 2017 and 2019, the maize total production worldwide was over 1.1 billion tons, while the area harvested exceeded 196 million ha [1]. According to the Ministry of Agriculture and Rural Development of Romania, after joining the European Union, national maize production doubled [2], meaning that Romania is recognized as the largest maize grower within the EU [3,4].

Carotenoids are important natural pigments in both the plant and animal kingdoms, as they are responsible for the yellow, orange, red and even blue colors of the tissues in which they appear. Humans and animals cannot synthesize carotenoids, so they are considered an essential component of the diet [5]. Maize with a high carotenoid content is an ideal source for carotenoid consumption in human and animal nutrition, as it usually contains lutein and zeaxanthin as major pigments [6]. Yellow and orange maize grains accumulate several carotenoids (α-carotene, β-carotene and β-cryptoxanthin) which can be transformed into vitamin A in the human body [7,8]. Lutein and zeaxanthin have antioxidant properties, and they accumulate in the retinal macula as they are also involved in vision process [9]; these carotenoids may help reduce the risk of certain types of cancer, particularly those of the breast and lung, and have a potential contribution to the prevention of heart disease and stroke [10], UV-induced skin damage, coronary heart disease [11], cataracts and macular degeneration [9,12,13].

Carotenoids with β-ring end groups taken from the diet act as precursors for the production of retinoids in animal cells and are essential components of human and animal nutrition. Provitamins A are linked to several essential functions in the body, such as reproductive functions, growth and immunity, while also presenting certain anti-cancer effects, preventing macular degeneration and reducing the risk of diabetes [14,15,16,17,18]. β-cryptoxanthin has been linked to a reduced risk of developing chronic diseases [19] and inflammatory disorders, such as rheumatoid arthritis and polyarthritis [20,21].

Although carotenoids are present in higher concentrations in fruits and vegetables, their presence in grains, such as maize, is of major concern and should be emphasized due to the importance of this cereal in the human diet worldwide, especially in regions with lower human development indexes [22]. Human consumption of plant metabolites such as carotenoids is below ideal, especially in poor regions, thus the improvement of the content in these components has a great importance. Unfortunately, most maize improvement programs are focused on obtaining higher productivity to the detriment of quality, but due to its importance in human nutrition, quality should be a priority. Due to the new possibilities of increasing the micronutrient levels of maize, biofortification programs for this plant have been of particular interest [18,23,24]. The vast majority of commercial maize varieties grown in the world have provitamin A carotenoids of around 2 µg/g [25]. The development of crops with high carotenoid content can help alleviate vitamin A deficiency in the poor regions of the world, as well as other nutritional and health problems worldwide [26].

The reported carotenoid contents in different landraces and inbred lines are highly variable (Table 1), depending mainly on the studied germplasm.

There are three main approaches used for maize biofortification: conventional, transgenic and agronomic [33]. Biofortified maize genotypes have been developed through conventional breeding programs that produce yellow and orange kernels. In order to improve the carotenoid content of maize, the selection of valuable genotypes can be used, followed by the choice of parents with a high carotenoid content for the creation of new hybrids. Another breeding method could be using parental lines with complimentary carotenoid profiles for crosses in order to increase the concentrations of both provitamin A and non-provitamin A carotenoids [29].

Marker-assisted selection was also used in order to improve the carotenoid content of some maize genotypes, by identifying the presence of favorable alleles crtRB1 and lcyE, followed by introgression of the genes through backcrosses [8,34,35,36,37,38,39,40,41]. These studies used the backcross method, but for a smaller number of generations than were used in the present study. Significant improvements in carotenoid content have been identified in both introgressed inbred lines and hybrids obtained through their use, compared to the initial genotypes. The method of introgression of favorable genes for carotene content has also been used successfully in the case of sweet corn [42,43].

Another possibility for improving the content of carotenoids could be the use of transgenic organisms [44,45,46] as a result of the success of Golden rice, but due to the European laws and concerns regarding biosafety, other methods of improving this content should be used.

Some research suggests that agronomic biofortification can also be used, as it offers a temporary micronutrient increase in the soil through fertilizers and is useful in increasing the micronutrients absorbed directly by the plant [47].

The discovery of cytoplasmic male sterility, but also in-depth research on the different types of cytoplasm, have opened new research areas, hence several studies were initiated on cytoplasmic diversification, extrachromosomal heredity and the influence of the cytoplasm on the transmission of some traits of agronomic interest in hybrids. The isonuclear lines offer the possibility for genetic studies regarding the cytoplasm and nucleus, but also for interactions between the two, increasing the diversity of the germplasm used in the vegetal production [48,49,50,51,52].

Isonuclear inbred lines are created by crossing a maternal line with a paternal line, followed by back-crossing for several generations with the paternal line (nucleus donor), until the nucleus of the donor line is transferred to the new cytoplasm [52].

The study of isonuclear inbred lines was initiated out of a desire to clarify whether the origin of the cytoplasm positively or negatively influences the traits of the cobs, plants, grains, and some trait of agronomic interest. Several studies have been conducted to examine the differences between the isonuclear lines regarding production and cob traits [50,52,53], vegetative traits [48,50,54,55], biochemical composition [56,57], pest and diseases resistance [58,59], but not in terms of carotenoid content. Most studies on maize isonuclear lines refer to cytoplasmic male sterility and not to fertile genotypes.

The main objectives of the isoline breeding program were to improve the genetic basis of the inbred lines from the maize breeding collection of the Agricultural Research and Development Station Turda, and also to study the influence of the cytoplasm on several traits. The aim of this study is to establish the influence of some fertile cytoplasm on the carotenoid content in several inbred lines and hybrids.

## 2. Results and Discussion

The carotenoid content is of great interest for the improvement of maize due to the importance of carotenoids in human and animal nutrition. The involvement of cytoplasmic actions or cytoplasmic interactions with the nucleus in the determinism of these traits could mean that cytoplasmic diversification can be used as a method to improve the carotenoid content of maize grains.

**Figure 1:**
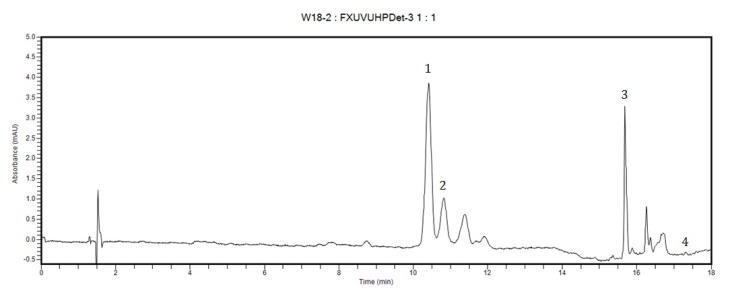
HPLC chromatogram of a representative genotype containing lutein and β-cryptoxanthin as major carotenoids. Peak IDs: 1—lutein, 2—zeaxanthin, 3—β-cryptoxanthin, 4—β-carotene.

The carotenoid content of maize grains from the studied genotypes revealed significant differences; such differences were also observed in chromatographic profiles obtained by high performance liquid chromatography (HPLC). HPLC analysis emphasized that lutein is the major carotenoid in most hybrids, while in a smaller number of cases β-cryptoxanthin was the major carotenoid; Figure 1 reveals the HPLC fingerprint for a representative genotype in which both lutein and β-cryptoxanthin are the major carotenoids, while containing only traces of β-carotene.

### 2.1. Cytoplasm Influence on Isonuclear Lines

Cytoplasmic actions attributed to cytoplasm TC221 resulted in significant decreases in the percentage of zeaxanthin in inbred lines, on average by 0.18 µg/g compared to the original line (Table 2). However, the same cytoplasm led to statistically significant increases for the β-carotene content of the lines, the increase compared to the original line being of 0.09 µg/g. The β-carotene content of inbred lines was also improved by using the cytoplasm TB329 to create isonuclear lines, the average increase being 0.12 µg/g.

Although the general combining ability of lines does not indicate a significant involvement of cytoplasm in the genetic determinism of carotenoid content, cytoplasm may play an important role in increasing or decreasing the percentage of these carotenoids, through actions due to cytoplasm and nucleus interaction (specific combining ability).

### 2.2. Cytoplasm × Nucleus Influence on Isonuclear Lines

Table 3 presents the results regarding the influence of the interaction between the cytoplasm and nucleus on carotenoid content of the studied inbred lines.

Following the interaction of TC 209 with the four sources of cytoplasm, some changes in carotenoid content were observed. Interaction with cytoplasm T248 resulted in significant increases in total carotenoid (+1.91 µg/g) and lutein (+1.64 µg/g) content, while the use of cytoplasm TC177 resulted in increases in zeaxanthin content with +0.47 µg/g. Cytoplasm TB329 increased the β-carotene content by 0.26 µg/g, the value for isoline representing 199% of the control value.

The original TC316 line had high per se values for carotenoid content, and by using the cytoplasm TC221, changes were seen for all the targeted carotenoids, with negative differences for total carotenoids, lutein and zeaxanthin, while β-cryptoxanthin and β-carotene increased statistically significantly. The decrease of −1.15 µg/g recorded for zeaxanthin is the largest for this trait, with the isoline value representing only 64% of the control. The β-cryptoxanthin content of isoline TC316 (cyt TC221) increased with +0.51 µg/g compared to the original line; the β-carotene content of this line was positively influenced by all four cytoplasms, the increases compared with the original line being between 0.13 and 0.36 µg/g, and the content representing 130–184% of the original line.

The content of total carotenoids, lutein, zeaxanthin and β-carotene of the TB367 line was positively influenced by the use of the cytoplasm TB329. The increase in total carotenoids, 2.34 µg/g, is the highest in the whole experience. Three of the four cytoplasms (TB329, TC177 and TC221) positively influenced the β-carotene content of TB367 line, the increases being between 0.28 and 0.43 µg/g, and the absolute values of the isonuclear lines representing 143–165% of the value of the original line.

In the case of line D105, by diversifying the cytoplasm, the β-carotene content was completely lost, with the losses of 0.31 µg/g being very significant. For the other carotenoids, no significant differences were identified due to the actions of the nuclear–cytoplasmic interaction.

### 2.3. Cytoplasm Influence on Hybrids

Change in the cytoplasm had a slight influence on the carotenoid content, but significant differences were recorded for lutein and β-cryptoxanthin when the maternal form of the hybrid used the cytoplasm TC221, compared with the original genotype (Table 4). The lutein content also increased significantly with the use of TB329 cytoplasm. Although the cytoplasm did not have a significant influence on the carotenoid content of maize grains, the differences between hybrids using the parental form with the modified cytoplasm and the original ones may be due to the interaction between the cytoplasm and maternal nucleus or even the interaction between the cytoplasm, nucleus and paternal line (tester).

### 2.4. Cytoplasm × Nucleus Influence on Hybrids

Table 5 highlights the results on the interaction between cytoplasm and nucleus for the carotenoid content of the studied hybrids. Each value represents the average of the four hybrids resulting from crossing each line with the four testers. Hybrids resulting from crossing the original maternal line and testers were used as a control for isoline × tester hybrids.

The genetic determinism of the transmission of total carotenoid content in maize is influenced by the lines crossed for the creation of the new hybrid, so it can be observed that for some hybrids, the carotenoid content was not modified by changing the cytoplasm of the maternal line, while for other hybrids the differences were statistically significant. There is a very significant negative influence of cytoplasms TC177 and TC221 on hybrids using TC316 as a maternal line (−2.29 µg/g and −1.93 µg/g), while the same cytoplasms caused very significant increases for hybrids with mother line D105 (+1.86 µg/g and +2.06 µg/g). It should be noted, however, that hybrids using the original TC316 as a maternal line had a higher total carotenoid content, while line D105 transmitted a lower total carotenoid content. Additionally, due to this fact, the other two cytoplasms, T248 and TB329, brought significant improvements for hybrids with the D105 line.

The lutein content increased in hybrids using the maternal lines TC243 (cytTC221) (+1.10 µg/g) and TB367(cytTC221) (+0.58 µg/g). The lutein content of hybrids obtained using the maternal TC243 line was also positively influenced by the use of cytoplasm TB329 (+1.04 µg/g).

The zeaxanthin content for maize hybrids resulted from crossing TC 316 line and the testers decreased when cytTC177 (−0.50 µg/g) and cytTC221 (−0.44 µg/g) lines were used for cytoplasmic diversification. However, D105 (cyt TC221) had a beneficial effect on the zeaxanthin of hybrids when used as maternal line.

The β-cryptoxanthin content of the studied hybrids was influenced by the change in the maternal cytoplasm, and both cases of both decreases in this carotenoid and statistically significant increases were encountered. The interaction of the maternal line TC209 with the cytoplasms TB329 and TC177 showed, in the case of hybrids, significant negative differences of −0.34 and −0.30 µg/g. The use of TB329 cytoplasm for the diversification of the maternal lines TC243 and D105 led to significant increases for this carotenoid, the differences being +0.25 and +0.27 µg/g, respectively. We also noticed that cytoplasm TC221 led to increases in the β-cryptoxanthin content of the hybrids using TC316 and TC243 maternal lines by +0.28 and 0.50 µg/g, respectively. Very significant increases were also recorded in the case of maternal isoline D105 (cyt TC177), the differences between the hybrids and the control being of +0.43 µg/g.

### 2.5. Cytoplasm × Nucleus × Tester Influence on Hybrids

The interaction between the cytoplasm, nucleus and tester represents the specific combining ability of the hybrids for each trait. Statistically significant differences were observed, being attributed to this interaction for all studied traits.

The change in the cytoplasm of the maternal line for the studied hybrids led to changes in the percentage of total carotenoids, and significant differences were observed. For some hybrids, cytoplasmic diversification had a negative effect, with more cytoplasms causing a decrease in the total carotenoid content. Table 6 presents only the significant differences.

For the hybrid TC209 × TA367, change in the maternal line cytoplasm resulted in significant differences for three out of four cases: −3.56 (cytTB329), −3.05 (cytTC177) and −3.93 µg/g (cytTC221).

Higher decreases in the total carotenoids were found in the hybrid TC316 × TA367, for which all cytoplasms studied had a negative effect, the differences between the hybrids with maternal isoline and control being between −2.53 and −6.29 µg/g. The values for this group of hybrids represented between 61% and 84% of the measured value for the control.

Hybrids were found that exceeded the control values, with the increases being between +2.18 µg/g [TB367(cyt TC177) × TA367] and +3.97 µg/g [D105 (cyt TC221) × TC385 A]. The measured values for these hybrids represent 121% and 195%, respectively, compared to the control of each.

After analyzing the interactions between the cytoplasm, the nucleus and the tester, it was observed that in the case of the hybrid TC209 × TA367, three of the four cytoplasms significantly influenced the lutein content when used for cytoplasmic diversification of the maternal line (Table 7). However, these cytoplasms caused decreases between −1.45 and −2.26 µg/g in the lutein content. The hybrid TC209 × TC385A is noteworthy, since when the cytoplasm was diversified, the lutein increased by 2.13 µg/g (cyt TB329) and by 2.40 µg/g (cyt TC221).

For the hybrid D105 × TC385A, the use of the cytoplasm TC221 resulted in increases of +2.33 µg/g, which represents 189% of the control value. The lutein content from the hybrid TC316 (cyt TC177) × TA 367 represents only 70% of the measured content for the control, the difference being −2.07 µg/g.

The zeaxanthin content (Table 8) showed some differences due to the actions of the cytoplasm × nucleus × tester interaction; in some hybrids, with a change to the cytoplasm, differences between −1.38 and +0.75 µg/g were observed.

The zeaxanthin content for TC 316 x TA367 hybrid decreased significantly with the used of cytoplasm TC177 instead of the original one (−1.38 µg/g), where the value represents only 52% of the control value. The same cytoplasm significantly negatively influenced the hybrids TC209(cyt TC177) × TA367 (−0.49 µg/g) and TC243(cyt TC177) × TE356 (−0.50 µg/g).

The cytoplasm TB329 had a significantly negative influence on the hybrids TC209 (cytTB329) × TA367 (−0.83 µg/g) and TC316 (cytTB329) × TA367 (−0.87 µg/g).

The use of cytoplasm T 248 resulted in significant increases in zeaxanthin content in three hybrids: TC209(cyt T248) × TC344 (+0.75 µg/g), TB367(cyt T248) × TA367 (+0.49 µg/g) and D105(cyt T248) × TE356 (+0.52 µg/g).

The cytoplasmic actions attributed to TC221 led to both decreases in zeaxanthin and situations when its influence was positive; in the genetic determinism of this trait, the action of each cytoplasm × nucleus × tester interaction was different. For the hybrids of inbred lines TC209(cyt TC221) and TC316(cyt TC221), the action of the cytoplasm TC221 caused decreases in zeaxanthin content, while for the hybrids of TB367(cyt TC221) or D105(cyt TC221) lines, the experimental data indicated significant increases.

The β-cryptoxanthin content (Table 9) of hybrid maize grains could be modified by changing the cytoplasm of the maternal inbred line, as interactions were identified that caused both a decrease in this carotenoid and significant increases due to nuclear–cytoplasmic actions.

The hybrid TC209 × TC344 had the highest content of β-cryptoxanthin (among hybrids using the original cytoplasm of the maternal form), and with a change to the cytoplasm, in three out of four cases, the β-cryptoxanthin content decreased, and the values recorded representing between 63 and 80% from the control value. The cytoplasm TC221 had, in general, a positive influence on the β-cryptoxanthin content, the differences compared to the original hybrids being between +0.45 µg/g and +1.24 µg/g, except for the hybrid TC209 × TC344, for which the loss was very significant, −1.06 µg/g.

The hybrid D105 × TE356 had a low content of β-cryptoxanthin, but due to the involvement of cytoplasmic nuclear actions in the determinism of this trait, a significant increase was achieved by using three cytoplasms to create maternal isonuclear lines. The use of cytoplasms T248 and TB329 for the creation of maternal isonuclear lines led to an increase of +0.53 µg/g of β-cryptoxanthin, which almost doubles the content of this carotenoid. Actions attributed to the triple interaction resulted in significant increases of +0.79 µg/g β-cryptoxanthin for the hybrid D105 (cytTC177) × TE356, representing 234% of the control value.

For the case of β-carotene content (Table 10), the interaction between the cytoplasm, the nucleus and the tester has a significant influence on the genetic determinism of this trait. In the case of the hybrid D105 × TC344, β-carotene was missing, but by using the cytoplasm TB329 for the maternal genotype it was possible to introduce this carotenoid with significant increase of 0.55 µg/g. Additionally, the β-carotene content of the hybrid D105 × TE356 has a low level, of only 0.15 µg/g, but the use of two cytoplasms, T248 and TC177, resulted in increases of 0.41 µg/g and 0.34 µg/g, respectively, where these values represent 381% and 332%, respectively, of the control value. For some hybrids, the cytoplasm diversification decreased the β-carotene content, their values representing only 50% and 64.2%, respectively, of the control values [TC316(cytTC221) × TC385A] and TC316(cyt TC221) × TC344), respectively.

A number of valuable hybrids have been identified that surpassed controls by a higher carotenoid content (Table 11). The hybrids that exceeded the controls are part of the TC209, TC243, TB367 and D105 groups, but not of the TC316 group. This can be explained by the fact that both the line TC316 and the hybrids obtained with the original cytoplasm of this line have higher carotenoid content compared to the rest of the studied genotypes, so that no heterosis was achieved for any trait.

It has been observed that some hybrids that have exceeded their control for carotenoid content have also had a higher yield. Data regarding the production traits of hybrids have been presented in a previous paper by the authors [52]. Hybrids TC209(cytT248) × TC344 and TB367(cytT248) × TE356 were noted for having a higher carotenoid content and also exceeded controls by 9.2 q/ha and 14.4 q/ha, respectively.

## 3. Materials and Methods

### 3.1. Biological Material and Experimental Design

**Figure 2:**
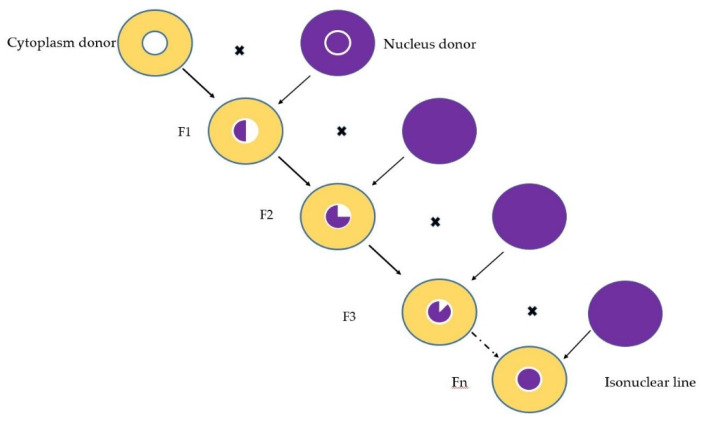
Nucleus transfer into a different cytoplasm through backcross in order to create isonuclear lines.

Out of the desire to avoid the genetic vulnerability resulting from the uniformity of the cytoplasms of inbred lines, as well as to find new sources to increase the heterotic capacity, in 1992, at the Agricultural Research and Development Station Turda, the transfer of the nucleus of some elite lines was initiated (inbred lines with good general combining ability) on several fertile cytoplasm sources. For the nucleus transfer, the backcross method was used for 10 generations. The nucleus of five elite inbred lines was transferred to four fertile cytoplasmic sources. The method of creating isonuclear lines is shown in Figure 2. The nucleus donor lines are TC209, TC316, TC243, TB367 and D105, while the cytoplasm sources used are T248, TB329, TC177 and TC221. Thus, the result was 20 isonuclear lines, and together with the 5 original inbred lines, they were studied in a 25 genotypes experimental crop.

The 25 inbred lines were crossed with 4 testers, to study the genetic determinism involved in the transmission of traits. The testers used are elite inbred lines TA367, TC344, TC385A and TE356. Following the crossing of the maternal genotypes with the four testers, 100 maize hybrids resulted.

In choosing the biological material, its genetic diversity was taken into account (Table 12), in order to obtain a heterosis following the crossing of inbred lines. Two of the nucleus donor lines (TC209 and TC243) are part of the heterotic group BSSS; TC316 line originates in the Lancaster group, and two nucleus donor lines are of the different flint types; Argentine flint (microsperm)—TB367, and European flint—D105. Regarding the cytoplasm donor lines, T248 belongs to Lancaster group and TB329 to Iodent group, while TC177 and TC221 are European flint type. The TA367 tester is the result of the crossing of the flint and Lancaster groups, and TC344 line has its origin in the crossing of the BSSS and flint groups. The TC385A tester line was included in the Lancaster germplasm group, and no single germplasm group was identified for the TE356 line.

All genotypes were studied for two years in the experimental field of the maize breeding laboratory, from the Agricultural Research and Development Station Turda, located in the Transylvanian Plateau, in the north-west of Turda, Cluj County, Romania, at an altitude between 345 and 493 m above sea level. The plant improvement fields are located on the upper terrace of the Aries river and they have a flat appearance, with frequent soil micro-depressions. The dominated soils are of the vertical clay-iluvial chernozem type. The most important biochemical indices have the following average values: humus content over 3.5%, mobile phosphorus content is 4.5 mg P2 05/100 g soil, and mobile potassium content is over 30 mg K_2_O/100 g sol. The soil reaction is neutral, being between 6.2 and 6.8 pH units.

The technology used was the same in both experimental years: hand sowing with the planter, herbicide, mechanical and manual tillage, manual harvesting. The precursor plant was autumn wheat and the plowing was carried out in autumn.

The experimental field was fertilized by applying a complex fertilizer of type N20: P20: K0—400 kg/ha, together with the preparation of the germination bed. Herbicide was performed pre-emergence with 1.5 L/ha, using S-metolachlor as the active substance (960 g/L) and post-emergence with 1.5 L/ha using tembotrione (44 g/L) and isoxadiphen-ethyl (22 g/L) as active substances.

The sowing density was 70,000 plants per hectare for inbred lines and 60,000 for hybrids. The cobs were covered at the beginning of their formation with a paper bag and were self-pollinated manually in order to avoid cross-pollination. Ten cobs were chosen from each plot in order to analyze their carotenoid content.

Primary data regarding the climatic conditions were collected from Turda Meteorological Station, part of the North Transylvania Meteorological Center, longitude 23°4′ E, latitude 46°35′ N, 427 m altitude. The climate of the experimental area is temperate continental. July and August are the warmest months, with average multiannual temperatures (55 years) being 19.6 °C and 19.2 °C, respectively. The months with the highest rainfall are June and July, with multiannual values of 80.6 mm and 74.7 mm, respectively. In the first year during the grain formation, the temperatures exceeded the multiannual average (+1.3 °C in July and +2.9 °C in August) while the precipitations were reduced (−39 mm in July and −12 mm in August), negatively influencing the maize crop. The second year was favorable to maize crop, as the temperature was normal (+0.8 °C in July and +0.7 °C in August), while the rainfall exceeded the multiannual average (+68 mm in July and +28 mm in August).

The values obtained for each isoline were compared with the original inbred line, and in the case of hybrids, for each genotype with maternal form an isoline, the control was the hybrid with the original form of the maternal line.

### 3.2. Materials

The carotenoid standards (lutein, zeaxanthin, β-cryptoxanthin and β-carotene) were from Sigma-Aldrich/Merck; acetonitrile, ethyl acetate and acetone were of HPLC grade (Merck, Darmstadt, Germany), while absolute ethanol for analysis was from Chimreactiv—Romania.

### 3.3. Quantification of Carotenoids

Representative maize grain samples of ~50 g were milled using a WZ-1 mill (Sandkiewicz Instruments—Poland), then 1 g of the resulting flour was weighed using an ABT 220-4M balance (Kern and Sohn, Germany); carotenoids were extracted using 50 mL of absolute ethanol, on a AM4 magnetic stirrer (Velp Scientifica—Italy) for 30 min, then the resulting suspension was filtered on a G3 frit, under vacuum.

For the quantification of major carotenoids, an aliquot from the extract was evaporated to dryness under vacuum at 40 °C in a Laborota 4010 rotary evaporator (Heidolph Instruments, Germany), then redissolved in 5 mL acetone, filtered through a 0.47 μm membrane filter and subjected to high performance liquid chromatography (HPLC).

**Figure 3:**
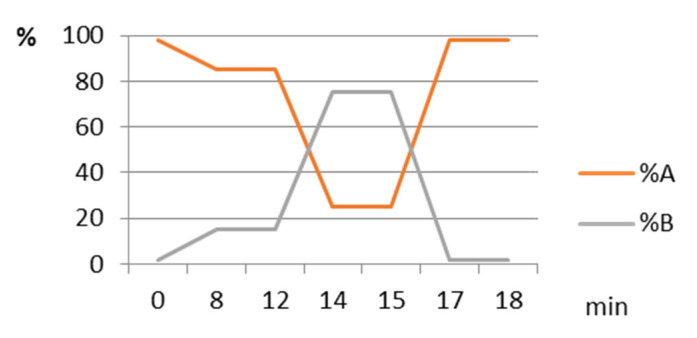
The gradient used in HPLC analysis. A: acetonitrile: H_2_O (9: 1, *w*/*w*), B: ethyl acetate.

HPLC analysis was accomplished using a Flexar system (Perkin Elmer, USA), consisting in two UHPLC pumps, a solvent degasser, an autoinjector, an UV-VIS detector, a column oven, a controller and a computer running Chromera software. Separations were monitored at 450 nm, using a Nucleosil 3-C_18_ column (Macherey Nagel) and a gradient involving ethyl acetate (A) and a mixture of 9:1 acetonitrile: water (Figure 3), at 25 °C; the flow rate was of 1 mL/min, the injected volumes were 5 μL, and the quantitative determinations were accomplished by the external standard method. Three replicates of each sample were analyzed and the mean values were reported.

All operations were carried out in reduced light, avoiding the samples’ exposure to more than 40 °C.

### 3.4. Data Processing

Data were analyzed using ANOVA PoliFact Soft, 2015. Fisher’s protected least significant difference (LSD) test was used to determine the significance of the differences among the genotypes using isonuclear lines and the controls (*p*-values 0.05, 0.01, and 0.001) for each experimental factor.

The values obtained for each isoline were compared with the original inbred line, and in the case of hybrids, for each genotype with maternal form an isoline, the control was the hybrid with the original form of the maternal line.

## 4. Conclusions

This study was carried out to identify whether the maternal cytoplasm could have an influence on the carotenoid content of some inbred lines and hybrids. Results show that the cytoplasm, but also the interaction between cytoplasm and nucleus or cytoplasm × nucleus × tester (for hybrids), can influence the content of total carotenoids, lutein, zeaxanthin, β-crypthoxanthin and β-carotene.

The cytoplasms did not have a spectacular influence on the carotenoid content of the inbred lines, but larger differences were attributed to the interaction between the cytoplasm and the nucleus. Regarding the values per se of the inbred lines, the cytoplasm sources significantly influenced only the β-carotene content, for the rest of the traits no significant differences due to the cytoplasm were identified.

The nuclear–cytoplasmic interaction is responsible for significant differences between the inbred lines regarding the β-carotene content, with the differences being positive or negative depending on the lines involved in the interaction. There have been some isolated cases when the cytoplasmic nuclear interaction influenced the rest of the traits. This means that the specific combining capacity must be taken into account, as the values of the general combining capacity are unrepresentative in this situation.

The transmission of carotene content in isonuclear lines is strongly influenced by the specifics of each interaction, as the specific combining ability for these traits have either a positive or a negative effect. Given the results obtained, it can be stated that changing the cytoplasm of inbred lines is a method that can be used to improve the per se values of the lines. However, the choice of biological material to be transformed should be taken into account so that the donor cytoplasms have the highest possible carotenoid content.

In the case of hybrids, cytoplasms influenced the lutein, zeaxanthin and β-cryptoxanthin content of maize grains. The cytoplasmic nuclear interaction also had a significant influence on the total carotenoids, but it was observed that for each nucleus, the interactions with the cytoplasms influenced a different number of traits.

For the cytoplasm × nucleus × tester interactions, significant differences were identified for all traits. It can be stated that the interaction has an important role in the heredity of the content of total carotenoids, lutein, zeaxanthin, β-cryptoxanthin and β-carotene.

Two hybrids: TC209(cytT248) × TC344 and TB367(cytT248) × TE356 had higher carotenoid content and also exceeded their controls regarding yield by 9.2 q/ha and 14.4 q/ha, respectively. It can be considered that some cytoplasms can be used to improve both the quality and the production.

Changing the cytoplasm of the maternal line may be a method of improving carotenoid content in maize hybrids. In choosing the genotypes to be used, some aspects must be taken into account, such as the high carotenoid content and the general good combination capacity, but the identification of the cross with the best specific combination ability must also be followed. In the attempt to obtain a superior quality of maize grains, the aspect of production should not be neglected, as that would make the improvement work more laborious.

## Figures and Tables

**Table 1:** Carotenoid content reported in studies regarding maize landraces and inbred lines (µg/g).

Varieties	Zeaxanthin	Lutein	β-Cryptoxanthin	β-Carotene	Reference
44 sweet and dent inbred lines	0.01–7.7	0.0–27.5	0.07–2.4	0.07–7.6	[27]
Over 1000 improved genotypes and 400 landraces	0.38–34.88	1.33–32.31	0.0–6.13	0.0–5.81	[28]
288 inbred lines (204 yellow lines)	0.76–43.9	0.0–31.0	0.16–10.8	0.07–13.6	[25]
430 Tropical adapted inbred lines	0.3–21.5	0.4–19	0.3–4.3	0.3–4.3	[29]
26 landraces	0.07–10.7	0.03–3.69	0.01–0.10		[15]
F2—different colors	0.09–11.8	1.1–19.1	0.01–5.4		[30]
127 inbred lines	2.77–14.88	0.01–7.22	1.65–9.71		[31]
22 landraces varieties and one commercial hybrid	1.85–26.95	3.50–35.30	0.30–13.85	0.30–6.10	[24]
2 landraces from 4 locations	0.05–14.90	0.12–24.99	0.08–8.83		[17]
477 lines (2014) and 496 lines (2016)	0.0–42.8	0.0–52.9	0.1–9.6		[32]

**Table 2:** Cytoplasm influence on carotenoid content for a group of isonuclear lines (µg/g)—ANOVA test.

Cytoplasm	Total Carotenoids	Lutein	Zeaxanthin	β-Cryptoxanthin	β-Carotene
	Mean ± SE	Mean ± SE	Mean ± SE	Mean ± SE	Mean ± SE
Original cyt	9.70 ± 0.78	5.37 ± 0.37	1.84 ± 0.17	0.77 ± 0.09	0.39 ± 0.04
Cyt T248	10.11 ± 0.80	5.37 ± 0.37	1.84 ± 0.14	0.74 ± 0.11	0.41 ± 0.06
Cyt TB329	10.13 ± 0.80	5.58 ± 0.37	1.87 ± 0.13	0.78 ± 0.10	0.50 ± 0.08 ***
Cyt TC177	9.89 ± 0.74	5.56 ± 0.35	1.79 ± 0.13	0.71 ± 0.10	0.41 ± 0.08
Cyt TC221	9.78 ± 0.64	5.35 ± 0.32	1.66 ± 0.08 ^0^	0.83 ± 0.14	0.47 ± 0.09 ***
LSD 5%	0.70	0.37	0.18	0.08	0.05
LSD 1%	0.93	0.50	0.24	0.11	0.07
LSD 0.1%	1.23	0.6	0.32	0.14	0.09

*** = Significant at 0.1% probability levels, positive values; ^0^ = Significant at 5% probability levels, negative values SE = Standard error.

**Table 3:** The influence of cytoplasm × nucleus interaction on the carotenoid content of the isonuclear lines (µg/g)—ANOVA test.

Cytoplasm	Total Carotenoids	Lutein	Zeaxanthin	β-Cryptoxanthin	β-Carotene
	Mean ± SE	Mean ± SE	Mean ± SE	Mean ± SE	Mean ± SE
**TC209**					
Original cyt	8.15 ± 0.31	4.67 ± 0.35	1.61 ± 0.14	0.74 ± 0.04	0.26 ± 0.03
T248	10.06 ± 0.36 *	6.31 ± 0.39 *	1.78 ± 0.06	0.67 ± 0.06	0.27 ± 0.02
TB329	9.23 ± 0.06	5.56 ± 0.25 *	1.44 ± 0.03	0.75 ± 0.04	0.52 ± 0.04 ***
TC177	8.92 ± 0.42	5.32 ± 0.35	2.09 ± 0.04 *	0.58 ± 0.05	0.25 ± 0.03
TC221	9.42 ± 0.40	5.43 ± 0.35	1.80 ± 0.09	0.62 ± 0.06	0.23 ± 0.02
**TC316**					
Original cyt	16.0 ± 0.42	8.1 ± 0.41	3.21 ± 0.30	1.53 ± 0.10	0.43 ± 0.05
T248	15.42 ± 0.65	7.01 ± 0.51 ^0^	2.99 ± 0.13	1.62 ± 0.13	0.76 ± 0.04 ***
TB329	15.78 ± 0.86	7.30 ± 0.49	2.85 ± 0.11	1.58 ± 0.14	0.80 ± 0.03 ***
TC177	15.32 ± 0.87	7.65 ± 0.32	2.70 ± 0.24 ^0^	1.53 ± 0.08	0.56 ± 0.03 *
TC221	14.41 ± 0.65 ^0^	7.26 ± 0.46 ^0^	2.06 ± 0.13 ^000^	2.04 ± 0.09 ***	0.69 ± 0.06 ***
**TC243**					
Original cyt	9.39 ± 0.42	5.58 ± 0.19	1.43 ± 0.07	0.66 ± 0.04	0.28 ± 0.02
T248	10.50 ± 0.75	6.13 ± 0.34	1.55 ± 0.10	0.69 ± 0.05	0.39 ± 0.04 *
TB329	9.08 ± 0.49	5.60 ± 0.52	1.34 ± 0.08	0.59 ± 0.03	0.26 ± 0.02
TC177	10.09 ± 0.47	5.96 ± 0.57	1.32 ± 0.11	0.60 ± 0.03	0.31 ± 0.01
TC221	9.19 ± 0.61	5.40 ± 0.47	1.26 ± 0.08	0.68 ± 0.04	0.38 ± 0.03
**TB367**					
Original cyt	8.91 ± 0.33	4.99 ± 0.27	1.29 ± 0.11	0.58 ± 0.06	0.65 ± 0.05
T248	9.82 ± 0.38	4.68 ± 0.21	1.42 ± 0.09	0.55 ± 0.05	0.63 ± 0.06
TB329	11.25 ± 0.27 **	6.40 ± 0.46 **	2.10 ± 0.05 ***	0.71 ± 0.07	0.93 ± 0.06 ***
TC177	9.32 ± 0.32	5.60 ± 0.32	1.25 ± 0.10	0.50 ± 0.04	0.95 ± 0.09 ***
TC221	9.66 ± 0.49	5.26 ± 0.23	1.32 ± 0.06	0.53 ± 0.03	1.08 ± 0.07 ***
**D105**					
Original cyt	6.06 ± 0.36	3.53 ± 0.34	1.68 ± 0.11	0.34 ± 0.02	0.31± 0.01
T248	4.74 ± 0.12	2.75 ± 0.09	1.47 ± 0.14	0.19 ± 0.01	0± 0 ^000^
TB329	5.34 ± 0.29	3.03 ± 0.24	1.61 ± 0.06	0.29 ± 0.02	0± 0 ^000^
TC177	5.82 ± 0.42	3.26 ± 0.20	1.58 ± 0.04	0.37 ± 0.03	0± 0 ^000^
TC221	6.22 ± 0.39	3.38 ± 0.21	1.88 ± 0.14	0.28 ± 0.03	0± 0 ^000^
LSD 5%	1.56	1.56	0.41	0.18	0.11
LSD 1%	2.09	2.09	0.55	0.24	0.15
LSD 0.1%	2.74	2.74	0.72	0.32	0.19

*, **, *** = Significant at 5%, 1%, and 0.1% probability levels, positive values; ^0^, ^000^ = Significant at 5% and 0.1% probability levels, negative values. SE ± = Standard error.

**Table 4:** Cytoplasm influence on the carotenoid content of the hybrids (µg/g)—ANOVA test.

Cytoplasm	Total Carotenoids	Lutein	Zeaxanthin	β-Cryptoxanthin	β-Carotene
	Mean ± SE	Mean ± SE	Mean ± SE	Mean ± SE	Mean ± SE
Original cyt	10.21 ± 0.44	5.14 ± 0.18	1.68 ± 0.08	1.27 ± 0.05	0.67 ± 0.04
Cyt T248	10.54 ± 0.45	5.32 ± 0.20	1.78 ± 0.08	1.34 ± 0.06	0.68 ± 0.04
Cyt TB329	10.40 ± 0.40	5.41 ± 0.16 *	1.63 ± 0.07	1.30 ± 0.05	0.61 ± 0.04
Cyt TC177	9.87 ± 0.38	5.11 ± 0.16	1.57 ± 0.08 ^0^	1.25 ± 0.06	0.61 ± 0.05
Cyt TC221	10.38 ± 0.30	5.42 ± 0.14 *	1.68 ± 0.06	1.48 ± 0.05 ***	0.63 ± 0.04
LSD 5%	0.47	0.26	0.10	0.10	0.07
LSD 1%	0.62	0.34	0.14	0.13	0.10
LSD 0.1%	0.80	0.44	0.17	0.16	0.12

*, *** = Significant at 5% and 0.1% probability levels, positive values; ^0^ = Significant at 5%, probability levels, negative values. SE = Standard error.

**Table 5:** The influence of cytoplasm × nucleus interaction on the carotenoid content of the hybrids (µg/g)—ANOVA test.

Cytoplasm	Total Carotenoids	Lutein	Zeaxanthin	β-Cryptoxanthin	β-Carotene
	Mean ± SE	Mean ± SE	Mean ± SE	Mean ± SE	Mean ± SE
**TC209**					
Original cyt	12.43 ±1.03	6.73 ± 0.45	1.81 ± 0.13	1.59 ± 0.08	0.60 ± 0.05
T248	13.40 ±1.17	7.12 ± 0.56	1.97 ± 0.18	1.71 ± 0.10	0.66 ± 0.07
TB329	12.68 ±1.07	7.04 ± 0.34	1.70 ± 0.17	1.25 ± 0.11 ^00^	0.49 ± 0.05
TC177	12.19 ± 0.73	6.56 ± 0.28	1.84 ± 0.17	1.29 ± 0.12 ^00^	0.46 ± 0.07
TC221	11.41 ± 0.63	6.20 ± 0.27	1.64 ± 0.15	1.47 ± 0.10	0.60 ± 0.06
**TC316**					
Original cyt	14.17 ± 0.70	6.32 ± 0.21	2.45 ± 0.17	1.89 ± 0.11	1.00 ± 0.07
T248	13.57 ± 0.57	5.98 ± 0.22	2.36 ± 0.14	1.90 ± 0.13	0.84 ± 0.05
TB329	13.31 ± 0.66	5.97 ± 0.24	2.26 ± 0.20	1.89 ± 0.12	0.76 ± 0.07
TC177	11.89 ± 0.81 ^000^	5.52 ± 0.31 ^0^	1.95 ± 0.18 ^000^	1.78 ± 0.14	0.85 ± 0.06
TC221	12.24 ± 0.60 ^000^	5.76 ± 0.23	2.01 ± 0.13 ^000^	2.17 ± 0.14 **	0.84 ± 0.10
**TC243**					
Original cyt	8.71 ± 0.49	4.30 ± 0.12	1.16 ± 0.09	1.07 ± 0.11	0.70 ± 0.08
T248	8.29 ± 0.52	4.75 ± 0.23	1.15 ± 0.13	1.11 ± 0.16	0.67 ± 0.10
TB329	9.82 ± 0.50 *	5.34 ± 0.23 ***	1.25 ± 0.10	1.32 ± 0.12 *	0.62 ± 0.08
TC177	7.86 ± 0.65	4.66 ± 0.32	0.94 ± 0.09	1.00 ± 0.14	0.54 ± 0.11
TC221	9.64 ± 0.56	5.40 ± 0.24 ***	1.26 ± 0.10	1.57 ± 0.12 ***	0.58 ± 0.08
**TB367**					
Original cyt	9.77 ± 0.67	4.98 ± 0.30	1.64 ± 0.13	1.12 ± 0.13	0.80 ± 0.06
T248	10.31 ± 0.77	5.08 ± 0.35	1.77 ± 0.16	1.17 ± 0.15	0.93 ± 0.08
TB329	9.12 ± 0.59	4.77 ± 0.23	1.53 ± 0.12	1.09 ± 0.13	0.83 ± 0.11
TC177	9.58 ± 0.76	4.76 ± 0.31	1.56 ± 0.13	1.07 ± 0.15	0.87 ± 0.14
TC221	10.60 ± 0.75	5.56 ± 0.41 *	1.72 ± 0.15	1.32 ± 0.12	0.86 ± 0.11
**D105**					
Original cyt	5.96 ± 0.32	3.39 ± 0.17	1.35 ± 0.11	0.68 ± 0.09	0.10 ± 0.05
T248	7.13 ± 0.54 *	3.69 ± 0.21	1.64 ± 0.18 *	0.83 ± 0.12	0.29 ± 0.05 *
TB329	7.09 ± 0.38 *	3.93 ± 0.19	1.41 ± 0.12	0.94 ± 0.10 *	0.34 ± 0.06 **
TC177	7.82 ± 0.57 ***	4.05 ± 0.24	1.54 ± 0.15	1.10 ± 0.10 ***	0.33 ± 0.09 **
TC221	8.02 ± 0.30 ***	4.19 ± 0.21	1.78 ± 0.11 ***	0.86 ± 0.13	0.28 ± 0.02 *
LSD 5%	1.06	0.58	0.23	0.22	0.16
LSD 1%	1.36	0.76	0.30	0.28	0.22
LSD 0.1%	1.79	0.98	0.39	0.37	0.28

*, **, *** = Significant at 5%, 1%, and 0.1% probability levels, positive values; ^0^, ^00^, ^000^ = Significant at 5%, 1%, and 0.1% probability levels, negative values. SE = Standard error.

**Table 6:** The influence of cytoplasm × nucleus × tester interaction on some hybrids’ content in total carotenoids (µg/g)—ANOVA test.

Hybrid	Original Cyt	Cyt T248	Cyt TB329	Cyt TC177	Cyt TC221
	Mean ± SE	Mean ± SE	Mean ± SE	Mean ± SE	Mean ± SE
TC209 × TA367	14.38 ± 1.42		10.82 ± 0.80 ^00^	11.33 ± 1.09 ^00^	10.45 ± 0.75 ^000^
TC209 × TC344	14.59 ± 1.20	17.36 ± 0.97 *			11.33 ± 1.01 ^00^
TC209 × TC385A	7.09 ± 0.57			9.22 ± 0.72 *	9.55 ± 0.79 *
TC316 × TA367	16.07 ± 1.58	13.21 ± 1.09 ^00^	13.54 ± 1.06 ^0^	9.78 ± 0.52 ^000^	12.66 ± 1.06 ^00^
TC316 × TA385A	11.75 ± 1.09			9.00 ± 0.79 ^0^	
TC316 × TC344	14.46 ± 1.09				11.78 ± 1.07 ^0^
TC243 × TA367	8.90 ± 0.75		11.30 ± 0.26 *		
TC243 × TC385A	6.88 ± 0.55			−4.66 ± 0.38 ^0^	
TB367 × TA367	10.61 ± 0.82			12.79 ± 0.50 *	
TB367 × TC344	9.67 ± 0.85				13.17 ± 1.03 **
TB367 × TE356	12.43 ± 1.17		10.23 ± 0.96 ^0^		
D105 × TA367	5.91 ± 0.25			9.47 ± 0.68 **	8.66 ± 0.65 *
D105 × TC385A	4.19 ± 0.21				8.16 ± 0.78 ***
D105 × TE356	7.15 ± 0.11			9.83 ± 0.81 *	
LSD 5%	2.11				
LSD 1%	2.78				
LSD 0.1%	3.58				

*, **, *** = Significant at 5%, 1%, and 0.1% probability levels, positive values; ^0^, ^00^, ^000^ = Significant at 5%, 1%, and 0.1% probability levels, negative values. SE = Standard error.

**Table 7:** The influence of cytoplasm × nucleus × tester interaction on some hybrids’ lutein content (µg/g)—ANOVA test.

Hybrid	Original Cyt	Cyt T248	Cyt TB329	Cyt TC177	Cyt TC221
	Mean ± SE	Mean ± SE	Mean ± SE	Mean ± SE	Mean ± SE
TC209 × TA367	8.14 ± 0.58		5.88 ± 0.50 ^000^	6.69 ± 0.35 ^0^	6.03 ± 0.54 ^000^
TC209 × TC344	7.20 ± 0.65				5.47 ± 0.35 ^000^
TC209 × TC385A	4.20 ± 0.38		6.33 ± 0.49 ***		6.60 ± 0.57 ***
TC209 × TE356	7.37 ± 0.43	8.64 ± 0.74 *			
TC316 × TA367	6.95 ± 0.50			4.88 ± 0.34 ^000^	
TC316 × TC385A	6.61 ± 0.47			4.74 ± 0.35 ^00^	5.29 ± 0.45 ^0^
TC243 × TA367	4.30 ± 0.21		6.24 ± 0.27 **	5.80 ± 0.51 *	5.74 ± 0.39 *
TC243 × TC344	4.28 ± 0.13				5.54 ± 0.50 *
TC243 × TE356	4.50 ± 0.27		5.69 ± 0.31 *		5.81 ± 0.52 *
TB367 × TC344	4.83 ± 0.36				7.09 ± 0.66 ***
D105 × TA367	3.34 ± 0.15			5.01 ± 0.42 **	
D105 × TC385A	2.62 ± 0.21				4.59 ± 0.40 ***
LSD 5%	1.15				
LSD 1%	1.52				
LSD 0.1%	1.96				

*, **, *** = Significant at 5%, 1%, and 0.1% probability levels, positive values; ^0^, ^00^, ^000^ = Significant at 5%, 1%, and 0.1% probability levels, negative values. SE = Standard error.

**Table 8:** The influence of cytoplasm × nucleus × tester interaction on some hybrids’ zeaxanthin content (µg/g)—ANOVA test.

Hybrid	Original Cyt	Cyt T248	Cyt TB329	Cyt TC177	Cyt TC221
	Mean ± SE	Mean ± SE	Mean ± SE	Mean ± SE	Mean ± SE
TC209 × TA367	2.18 ± 0.19		1.35 ± 0.11 ^00^	1.69 ± 0.18 ^0^	1.42 ± 0.13 ^00^
TC209 × TC344	1.99 ± 0.11	2.74 ± 0.25 **			
TC316 × TA367	2.88 ± 0.23		2.01 ± 0.18 ^000^	1.5 ± 0.13 ^000^	1.96 ± 0.13 ^000^
TC316 × TC344	2.42 ± 0.22				1.92 ± 0.08 ^0^
TC243 × TE356	1.67 ± 0.10			1.17 ± 0.08 ^0^	
TB367 × TA367	1.63 ± 0.16	2.12 ± 0.21 *			
TB367 × TC344	1.61 ± 0.13				2.17 ± 0.15 *
D105 × TA367	1.03 ± 0.09				1.59 ± 0.15 *
D105 × TC385A	0.93 ± 0.09				1.64 ± 0.16 **
D105 × TE356	1.97 ± 0.08	2.49 ± 0.20 *			
LSD 5%	0.46				
LSD 1%	0.61				
LSD 0.1%	0.78				

*, ** = Significant at 5% and 1%, probability levels, positive values; ^0^, ^00^, ^000^ = Significant at 5%, 1%, and 0.1% probability levels, negative values. SE = Standard error.

**Table 9:** The influence of cytoplasm × nucleus × tester interaction on some hybrids’ β-cryptoxanthin content (µg/g)—ANOVA test.

Hybrid	Original Cyt	Cyt T248	Cyt TB329	Cyt TC177	Cyt TC221
	Mean ± SE	Mean ± SE	Mean ± SE	Mean ± SE	Mean ± SE
TC209 × TA367	1.31 ± 0.16		0.58 ± 0.17 ^00^	0.43 ± 0.24 ^000^	
TC209 × TC344	3.10 ± 0.15		2.48 ± 0.22 ^00^	1.95 ± 0.15 ^000^	2.04 ± 0.19 ^000^
TC209 × TE356	1.13 ± 0.21			1.68 ± 0.17 *	1.68 ± 0.15 *
TC316 × TC344	2.78 ± 0.18		3.28 ± 0.13 *		3.23 ± 0.20 *
TC316 × TC385A	1.40 ± 0.16				1.94 ± 0.20 *
TC316 × TE356	1.95 ± 0.20		1.41 ± 0.27 ^0^		
TC243 × TA367	0.97 ± 0.17		1.45 ± 0.15 *		1.49 ± 0.18 *
TC243 × TC344	1.91 ± 0.19				3.15 ± 0.29 ***
TB367 × TC344	1.84 ± 0.23				2.73 ± 0.16 ***
D105 × TE356	0.59 ± 0.18	1.12 ± 0.15 *	1.12 ± 0.13 *	1.38 ± 0.15 ***	
LSD 5%	0.43				
LSD 1%	0.57				
LSD 0.1%	0.73				

*, *** = Significant at 5% and 0.1% probability levels, positive values; ^0^, ^00^, ^000^ = Significant at 5%, 1%, and 0.1% probability levels, negative values. SE = Standard error.

**Table 10:** The influence of cytoplasm × nucleus × tester interaction on some hybrids β-carotene content (µg/g)—ANOVA test.

Hybrid	Original Cyt	Cyt T248		Cyt TB329		Cyt TC177		Cyt TC221	
	Mean ± SE	Mean ± SE		Mean ± SE		Mean ± SE		Mean ± SE	
TC209 × TC344	1.0 ± 0.06					0.56 ± 0.09 ^00^			
TC209 × TE356	0.40 ± 0.04	0.77 ± 0.05 *							
TC316 × TC344	1.06 ± 0.06							0.68 ± 0.12 ^0^	
TC316 × TC385A	0.76 ± 0.11							0.37 ± 0.07 ^0^	
TC316 × TE356	1.40 ± 0.14			0.82 ± 0.09 ^000^					
TC243 × TA367	0.65 ± 0.09					1.08 ± 0.07 *			
TC243 × TE356	1.28 ± 0.11			0.85 ± 0.10 ^0^		0.69 ± 0.08 ^000^		0.68 ± 0.04 ^000^	
D105 × TC344	0 ± 0			0.55 ± 0.07 **					
D105 × TE356	0.15 ± 0.01	0.56 ± 0.06 *				0.49 ± 0.04 *			
LSD 5%	0.33								
LSD 1%	0.43								
LSD 0.1%	0.56								

*, ** = Significant at 5% and 1% probability levels, positive values; ^0^, ^00^, ^000^ = Significant at 5%, 1%, and 0.1% probability levels, negative values. SE = Standard error.

**Table 11:** Hybrids that exceeded the control (the hybrid using the original cytoplasm) for the targeted carotenoids (µg/g)—ANOVA test.

Hybrid	Total Carotenoids	Lutein	Zeaxanthin	β-Cryptoxanthin	β-Carotene
	Mean ± SE	Mean ± SE	Mean ± SE	Mean ± SE	Mean ± SE
**TC209 × TC344 (control)**	**14.59** ± 1.20	**7.2** ± 0.65	**1.99** ± 0.11	**3.1** ± 0.15	**1.0** ± 0.06
TC209(cyt T248) × TC344	17.36 ± 0.97 *	8.24 ± 0.43	2.75 ± 0.25 **	3.4 ± 0.12	1.03 ± 0.07
**TC209 × TC385A (control)**	**7.09** ± 0.57	**4.2** ± 0.38	**1.05** ± 0.11	**0.84** ± 0.21	**0.33** ± 0.03
TC209(cytTC221) × TC385A	9.55 ± 0.79 *	6.6 ± 0.57 ***	1.12 ± 0.08	1.04 ± 0.18	0.44 ± 0.03
**TC209 × TE356 (control)**	**13.64** ± 1.35	**7.37** ± 0.43	**2.03** ± 0.20	**1.13** ± 0.21	**0.4** ± 0.04
TC209(cyt T248) × TE356	15.71 ± 1.51	8.64 ± 0.74 *	2.05 ± 0.15	1.5 ± 0.16	0.74 ± 0.05 *
TC209(cyt TB329) × TE356	15.64 ± 1.37	8.32 ± 0.49	2.05 ± 0.21	1.16 ± 0.17	0.51 ± 0.02
TC209(cyt TC177) × TE356	13.86 ± 1.29	7.39 ± 0.53	2.31 ± 0.26	1.68 ± 0.17 **	0.62 ± 0.09
**TC243 × TA367 (control)**	**8.90** ± 0.75	**4.3** ± 0.21	**0.95** ± 0.08	**0.97** ± 0.17	**0.65** ± 0.09
TC243(cyt TB329) × TA367	11.3 ± 0.26*	6.24 ± 0.27 **	1.39 ± 0.12	1.45 ± 0.15 *	0.82 ± 0.08
**TC243 × TC344 (control)**	**8.12** ± 0.52	**4.28** ± 0.13	**0.97** ± 0.10	**1.91** ± 0.19	**0.56** ± 0.07
TC243(cyt TB329) × TC344	10.12 ± 0.70	4.9 ± 0.40	1.18 ± 0.04	1.98 ± 0.18	0.66 ± 0.09
TC243(cyt TC221) × TC344	10.12 ± 0.91	5.54 ± 0.50 *	1.13 ± 0.05	3.15 ± 0.29 ***	0.81 ± 0.05
**TB367 × TC344 (control)**	**9.67** ± 0.85	**4.83** ± 0.36	**1.61** ± 0.13	**1.84** ± 0.23	**0.68** ± 0.03
TB367(cyt TB329) × TC344	10.39 ± 0.63	5.46 ± 0.45	1.77 ± 0.13	1.95 ± 0.21	0.69 ± 0.07
TB367(cyt TC221) × TC344	13.16 ± 1.03 **	7.09 ± 0.66 ***	2.17 ± 0.15 *	2.73 ± 0.16 ***	0.83 ± 0.03
**TB367 × TE356 (control)**	**12.43** ± 1.17	**5.81** ± 0.50	**2.26** ± 0.18	**1.02** ± 0.20	**1.37** ± 0.08
TB367(cyt T248) × TE356	12.72 ± 1.08	6.31 ± 0.39	2.35 ± 0.23	1.16 ± 0.32	1.44 ± 0.09
**D105 × TA367 (control)**	**5.91** ± 0.25	**3.34** ± 0.15	**1.03** ± 0.09	**0.62** ± 0.21	**0.43** ± 0.03
D105(cyt TC177) × TA367	9.47 ± 0.68 **	5.01 ± 0.42 **	1.48 ± 0.29	1.04 ± 0.17	0.67 ± 0.10
**D105 × TC344 (control)**	**6.59** ± 0.49	**3.75** ± 0.29	**1.48** ± 0.09	**1.2** ± 0.17	**0** ± 0
D105(cyt TB329) × TC344	7.52 ± 0.66	3.97 ± 0.37	1.59 ± 0.16	1.31 ± 0.18	0.55 ± 0.07 ***
**D105 × TC385A (control)**	**4.19** ± 0.21	**2.62** ± 0.21	**0.93** ± 0.09	**0.27** ± 0.15	**0** ± 0
D105(cyt TB329) × TC385A	5.06 ± 0.28	3.06 ± 0.15	0.96 ± 0.06	0.47 ± 0.14	0.19 ± 0.02
D105(cyt TC177) × TC385A	5.11 ± 0.42	2.95 ± 0.22	1.04 ± 0.06	0.6 ± 0.19	0.08 ± 0.01
D105(cyt TC221) × TC385A	8.16 ± 0.78 ***	4.96 ± 0.40 ***	1.64 ± 0.16 **	0.68 ± 0.22	0.17 ± 0.01
**D105 × TE356 (control)**	**7.15** ± 0.11	**3.86** ± 0.36	**1.97** ± 0.08	**0.59** ± 0.18	**0.15** ± 0.01
D105(cyt T248) × TE356	9.1 ± 0.21	4.31 ± 0.10	2.49 ± 0.20 *	1.12 ± 0.15 *	0.56 ± 0.06 *
D105(cyt TC177) × TE356	9.82 ± 0.81 **	4.36 ± 0.30	2.35 ± 0.11	1.39 ± 0.15 ***	0.48 ± 0.04 *
LSD 5%	2.11	1.15	0.46	0.43	0.33
LSD 1%	2.78	1.52	0.61	0.57	0.43
LSD 0.1%	3.58	1.96	0.78	0.73	0.56

*, **, *** = Significant at 5%, 1%, and 0.1% probability levels, positive values; SE = Standard error.

**Table 12:** Cob traits of the inbred lines studied.

Inbred Line		Kernel Type	Kernel Color	Cob Color
Nucleus donor	TC 209	dent × flint	dark yellow	red
	TC 316	dent × flint	dark yellow	red
	TC 243	dent	yellow	white
	TB 367	flint	dark yellow	red
	D 105	flint	yellow	white
Cytoplasm donor	T 248	dent	yellow	red
	TB 329	dent	dark yellow	red
	TC 177	flint	dark yellow	white
	TC 221	flint	orange	white
Tester	TA 367	flint × dent	yellow	red
	TC 344	dent × flint	dark yellow	red
	TC 385A	dent	light tallow	white
	TE 356	dent × flint	dark yellow	red

## Data Availability

Not applicable.
